# Adsorption of Methylene Blue from Aqueous Solution Using Gelatin-Based Carboxylic Acid-Functionalized Carbon Nanotubes@Metal–Organic Framework Composite Beads

**DOI:** 10.3390/nano12152533

**Published:** 2022-07-23

**Authors:** Yang Zhang, Yanhui Li, Mingzhen Wang, Bing Chen, Yaohui Sun, Kewei Chen, Qiujv Du, Xinxin Pi, Yuqi Wang

**Affiliations:** 1 College of Mechanical and Electrical Engineering, Qingdao University, 308 Ningxia Road, Qingdao 266071, China; zy841876654lxm@outlook.com (Y.Z.); wmz8805618@163.com (M.W.); 2020020426@qdu.edu.cn (B.C.); syh1866038168@163.com (Y.S.); ckw980518@163.com (K.C.); pixinxin@qdu.edu.cn (X.P.); sdyuqi@126.com (Y.W.); 2 Laboratory of Fiber Materials and Modern Textile, The Growing Base for State Key Laboratory, Qingdao University, 308 Ningxia Road, Qingdao 266071, China; duqiuju@qdu.edu.cn

**Keywords:** adsorption, methylene blue, carbon nanotubes, metal–organic framework, gelatin

## Abstract

**Highlights:**

- A new gelatin composite was used to remove methylene blue.
- The adsorbent was composed of carbon nanotubes, a metal–organic framework and gelatin.
- The adsorbent had a simple preparation process and was friendly to the environment.
- The fixation of carbon nanomaterials with gelatin as the substrate avoided secondary pollution.
- Using carbon nanotubes as the intermediate improved the adsorption capacity.

**Abstract:**

A novel gelatin-based functionalized carbon nanotubes@metal–organic framework (F-CNTs@MOF@Gel) adsorbent was prepared by the green and simple method for the adsorption of methylene blue (MB). Cu-BTC (also known as HKUST-1) was selected as the MOF type. F-CNTs@Cu-BTC particles were fixed by gelatin, thus avoiding the secondary pollution of carbon nanomaterial particles to the environment. CNTs were used as the connecting skeleton to make more effective adsorption sites exposed on the surface of the internal pore structure of the adsorbent. In this paper, scanning electron microscopy (SEM), Fourier transform infrared spectroscopy (FTIR), powder X-ray diffraction (XRD), thermogravimetry (TGA) and BET analysis methods were used to characterize the new adsorbent. The effects of time, temperature, pH, dosage and initial concentration on the adsorption process were investigated by batch adsorption experiments. The adsorption mechanism was further analyzed by several commonly used kinetic and isotherm models, and the reliability of several fitting models was evaluated by the Akaike information criterion (AIC), Bayesian information criterion (BIC) and Hannan information criterion (HIC). After five regeneration experiments, the adsorbent still had 61.23% adsorption capacity. In general, the new adsorbent studied in this paper has an optimistic application prospect.

## 1. Introduction

With the rapid development of industrialization in the world, the problem of environmental pollution is becoming more and more serious. Among them, environmental pollution caused by textile, food packaging, printing, paper and other industrial fields is more obvious [1], mainly reflected in the sewage discharged by these types of factories containing a large number of dyes, which causes serious harm to the water resources, environment and ecosystem [2]. At present, the commonly used methods to remove pollutants in a water environment include chemical degradation, photocatalytic degradation, electrochemical removal, chemical flocculation, membrane filtration, physical adsorption, and so on [3,4]. Among them, the physical adsorption method has become one of the most popular methods in the field of water treatment due to its characteristics of simplicity, high efficiency, easy operation and good economy [5]. Common adsorbents used in physical adsorption include COFs [6], clay [7], hydrogel [8], activated carbon (AC), graphene oxide (GO), metal–organic frameworks (MOF), alginate, chitosan and their derivatives and complexes [9,10,11]. However, these traditional adsorbents have certain shortcomings, such as low adsorption capacity, poor recycling and secondary pollution caused by the adsorbents themselves.

Metal–organic frameworks (MOF) are a class of highly porous materials prepared by covalently bonding metal clusters or ionic and organic linkers [12]. Because MOFs have high porosity, large specific surface area and contain immobilized metal cations, MOFs are considered to be an ideal material for removing low-molecular-weight compounds from water and have been widely studied by scholars as an adsorbent [13]. Cu-BTC is a microporous open framework with a cubic symmetry octahedron structure, where Cu atoms are linked with oxygen atoms of eight tetracarboxylic acid units to form a dimer Cu wheel and each Cu-BTC ligand contains three dimer Cu wheels. [14,15]. Unfortunately, MOFs are usually in powder form, which is difficult to recycle and causes secondary contamination [16].

In order to overcome the difficult recovery of powder sorbents, porous macromolecular polymer materials to coat these powder sorbents can be used. For example, Pei et al. used gelatin (Gel)@Calcium alginate (CA) as a binder to cover the MOF, and the removal rate of MB by Gel-CA without the MOF was 43.6% (0.86 mg·g^−1^). Surprisingly, the removal rate reached 99.8% (1.99 mg·g^−1^) after the combination of the Gel-CA and MOF [17]. Moreover, Samaneh Saber-Samandari et al. used gelatin-coated carboxylated functionalized carbon nanotubes (F-CNTs) and magnetic nanoparticles of iron oxide (MNPs) to remove 96.1% (961 mg·g^−1^) of direct red (DR) and 76.3% (763 mg·g^−1^) of the MB [18]. Interestingly, these carbon nanomaterial adsorbents sometimes perform better than single adsorbents when combined with macromolecular polymers, suggesting that the two different adsorbents have a synergistic effect on the adsorption of pollutants. In general, the adsorption capacity of carbon powder monomer is better than that of the adsorbent after the powder and polymer composite because the specific surface area of powder is much larger than that of polymer. However, a large number of carbon nanomaterial particles that play a major adsorption role will be completely coated by macromolecular polymers, which will lead to the low utilization of carbon nanomaterials that play a major adsorption role. Faced with such problems, this study provided a solution to improve the problem to a certain extent.

In this work, in order to solve the problems mentioned above, we first grew Cu-BTC particles with the octahedral structure on F-CNTs with a slender structure and a stable performance according to the method of V. Jabbari and Zhang et al. [19,20]. F-CNTs@Cu-BTC (F-C) was formed in hydrothermal reactions where the carboxyl group (-COOH) on the surface of F-CNTs provided sites for Cu-BTC growth, thus forming a strong binding between F-CNTs and Cu-BTC. Subsequently, F-C was compounded with gelatin. Gelatin has favorable properties for the production of adsorbent composites due to its excellent gel-forming ability, low procurement cost, biodegradability and hydrophobicity [21]. Then, we used glutaraldehyde as a crosslinking agent to crosslink the molecular chains of gelatin to enhance their mechanical structure. The newly synthesized adsorbent with carbon nanotubes as the connecting frame exposed more carbon nanomaterial powders on the surface of the adsorbent to further increase the specific surface area of the adsorbent.

Methylene blue (MB), as a cationic organic dye, is often detected in natural water. MB can cause harm to human health, such as eye, respiratory, digestive and mental disorders [22]. Li et al. used Cu-BTC to adsorb simulated sewage solution containing Rhodamine B (RhB), MB and methyl orange (MO), and the removal rates of RhB, MB and MO were 47% (47 mg·g^−1^), 94% (94 mg·g^−1^) and 13% (13 mg·g^−1^), respectively. It showed that Cu-BTC had a selective and good adsorption effect for MB [14]. According to this conclusion, methylene blue was selected as the pollutant in this study.

In this study, the new composite material was prepared into a bead shape by dropping rate emulsification. The adsorption properties of F-CNTs@Cu-BTC@Gel (F-C-G) beads for MB were tested by batch adsorption experiments. In order to show that the addition of F-CNTs significantly improved the properties of the new adsorbent, we set Cu-BTC@Gel (C-G) composites without F-CNTs as the control group. The change of the maximum adsorption capacity (*q_m_*) before and after adding CNTs was from 88 mg·g^−1^ to 106 mg·g^−1^. Meanwhile, the adsorption capacities of the Cu-BTC, F-CNTs, F-C and Gel were tested. Fourier transform infrared spectroscopy (FTIR), scanning electron microscopy (SEM), powder X-ray diffraction (XRD), thermogravimetry (TGA) and BET analysis methods were used to characterize the new adsorbent by changing the temperature, time, pH, dosage and initial concentration of the adsorption process to determine the appropriate adsorption environment. Additionally, the experimental data were processed by three kinetic models and five isotherm models and thermodynamic analysis to further explore the adsorption capacity of the new adsorbent. Recently, the reliability of the adsorption model was evaluated by Akaike, Bayesian and Hannan information criterion (AIC, BIC, HIC), the modified AIC (AIC_C_) and Akaike weight (*w_i_*) [23,24]. Therefore, in this study, the reliability of the three models of adsorption kinetics and the five models of adsorption isotherms were judged by the above information criteria so as to make the experimental results more convincing. Meanwhile, the adsorption mechanism of MB was analyzed, and the regeneration experiment was carried out. Regeneration experiment results show that the adsorption capacity of the F-C-G bead after five times of regeneration is 61% of that of the original adsorbent.

## 2. Materials and Methods

### 2.1. Reagents and Materials

Benzene-1,3,5-tricarboxylic acid (H_3_BTC, purity ≥ 97%) was bought from Shanghai Hao Hong Biomedical Technology Co. Ltd., Shanghai, China. Multiwalled carbon nanotubes (MWCNTs, diameter 20–40 nm, length 5–15 μm, purity > 95%) were gained from Shenzhen Nanotech Port Co. Ltd. N, N-dimethylformamide (DMF, 98%) was purchased from Sinopharm Chemical Reagent Co. Ltd. (Shanghai, China). Gelatin was bought from Shanxi Tangyao Biological Technology Co. Ltd. (Shanxi, China). Copper nitrate trihydrate (Cu(NO_3_)_2_·3H_2_O, 99%) was acquired from Tianjin Ruijinte Chemical Co. Ltd., Tianjin, China. Concentrated sulfuric acid (H_2_SO_4_, 98 wt%) and Nitric acid (HNO_3_,65 wt%) were bought from Sinopharm Chemical Reagent Co. Ltd., Shanghai, China. All chemicals used in this experiment were of analytical grade and were used directly without further purification. All solutions were prepared with deionized water.

### 2.2. Preparation of F-CNTs

In this paper, carboxyl functional groups were added to the surface of the CNTs by the acid bath reflux method introduced by Li et al. [25]. The specific methods in this experiment are described as follows. An amount of 10 g of the CNTs were dispersed into a mixture of 320 mL concentrated sulfuric acid and 65 wt% nitric acids (3:1, v/v) and refluxed at 413 K for 20 min. After the reaction, it was diluted with deionized water and left for 24 h, then the supernatant was dumped. The above operation was repeated 4–5 times until the pH of the supernatant reached between 6 and 7. Finally, the prepared carboxylated functionalized carbon nanotubes (F-CNTs) were dried and collected for reserve.

### 2.3. Synthesis of F-CNTs@Cu-BTC

**Figure 1:**
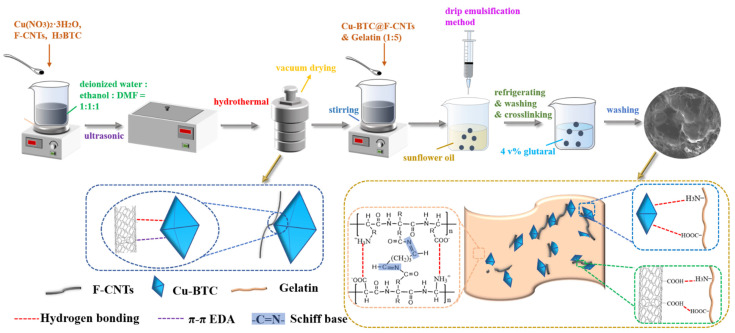
The preparation process of composite materials.

The synthesis method of the F-CNTs@Cu-BTC (F-C) composite was improved by combining the method given by V. Jabbari et al. [20] and Zhang et al. [19]. The specific synthesis process in this experiment was described as follows. An amount of 0.20 g F-CNTs, 1.50 g Cu(NO_3_)_2_·3H_2_O and 0.75 g H_3_BTC were placed in a solution of 70 mL ethanol:DMF:water = 1:1:1 (*v/v*), stirred for 2 h and fully dispersed by ultrasound for 20 min. The fully mixed solution was then transferred to a 100 mL (Teflon–stainless steel) autoclave. The hydrothermal reactor was placed in the oven at 373 K for 48 h. In this step, the carboxyl groups (-COOH) on the F-CNTs provided growth sites for the Cu-BTC. Moreover, the F-CNTs were closely bound to Cu-BTC by hydrogen bonding and π-π EDA (see Figure 1). After the reaction and cooling, the black solid was washed three times with ethanol and water. Finally, the black solid was dried at 393 K for 8 h to remove the excess DMF and ethanol from the surface of it. The preparation of Cu-BTC as a control group was similar to the above method, except that the F-CNTs were not added.

### 2.4. Synthesis of F-CNTs@Cu-BTC@Gel Beads

F-CNTs@Cu-BTC@Gel (F-C-G) beads were prepared on the basis of the method reported by Cesar Vinicius Toniciolli Rigueto et al. [21] and Pei et al. [17]. The preparation process in this study is shown in Figure 1, and the specific steps are described below. An amount of 0.8 g of prepared F-C powder and 4.0 g of gelatin (Gel) solid particles were placed in 50 mL of deionized water. F-C powder was evenly dispersed in gelatin solution by stirring at 323 K for 3 h. Then, using a syringe, the mixture was slowly added to 200 mL of sunflower oil, forming beads coated with an oil film. Subsequently, the beaker containing rubber beads and oil was put into the cold room at 278 K for 2 h in order to make the rubber beads solidify. The coagulated beads were filtered out and washed 5 times with a mixture of acetone:water = 1:1 to remove the oil on the surface of the beads. The beads were then placed in a beaker containing 4 v% glutaraldehyde solution and placed at room temperature for 24 h. The purpose of this was to make the macromolecular chains of gelatin fully crosslinked, thus making the gelatin-based composite more stable in structure. In this step, Cu-BTC and F-CNTs were connected to the amino (-NH_2_) and carboxyl (-COOH) groups in the gelatin molecule by hydrogen bonds. The Schiff base with strong force was formed after the gelatin molecules were crosslinked by glutaraldehyde [26]. There were also hydrogen bonding forces between amino (-NH_2_) and carboxyl (-COOH) groups in gelatin molecules (see Figure 1). The crosslinked beads were removed from the glutaraldehyde solution and washed 3 times with ethanol and deionized water to remove excess glutaraldehyde. Then, the washed beads were placed in a refrigerator at 255 K for 12 h and they were dried in a vacuum freeze-dryer. Finally, F-C-G beads were successfully obtained. The preparation process of C-G beads as a control group was similar to the above method, except that the F-C powder was replaced with Cu-BTC powder of the same quality.

### 2.5. Characterization

The surface morphology of the adsorbent was photographed by a field emission scanning electron microscope (SEM, Supra55, Zeiss, Germany, operated at a voltage of 15 kV). The specific surface area and pore size distribution of the adsorbent were measured by the BET specific surface area test method (Micromeritics ASAP2460-2 M, Mike, GA, USA). A Fourier transform infrared spectrometer (FT-IR, Tensor 27, Bruker, Germany) was used to analyze whether there were functional groups in the material in the wavenumber range 4000–400 cm^−1^. The thermogravimetric analyzer (TGA, Q5000, TA Instruments, New Castle, DE, USA) was used to analyze the samples, which were heated from 303 K to 973 K in N_2_ at a rate of 10 K·min^−1^. The crystal structure was verified by X-ray powder diffraction (XRD, Rigaku D/max 2500v/pc, Rigaku, Japan). During the whole experiment, the concentration of MB solution was measured by a UV–visible spectrophotometer (TU-1810, Beijing Purkinje General Instrument Co. Ltd., Beijing, China), and the wavelength used to measure MB was 664 nm.

### 2.6. Adsorption Experiments

The adsorption performance of the F-C-G beads for MB was tested by batch adsorption experiments. Meanwhile, the equilibrium adsorption capacities of Cu-BTC, F-CNTs, F-C, Gel and C-G were tested to explore the differences in their adsorption capacities. Control experiment parameters included temperature, time, pH, the dosage of adsorbent and the initial concentration of the MB. All samples oscillated at a constant speed of 190 rpm in a thermostatic water bath oscillator. In this study, we set 3 different temperatures (298 K, 308 K, 318 K) to explore the influence of temperature on the adsorption process, and the initial concentration of the MB was 40, 60, 80, 100 and 120 mg·L^−1^. In the experiments on the influence of pH on the adsorption process, the pH values were set from 2 to 11. In the experiment on the effect of the dose on the adsorption behavior, the dosage of the adsorbent was set at 2, 6, 10, 15 and 20 mg, and the concentration of the MB solution was 100 mg·L^−1^. In the time effect experiment, 250 mL of 100 mg·L^−1^ MB solution with 125 mg adsorbent was used. The concentration of the MB was measured at intervals of 2, 2, 4, 4, 8, 8, 16, 16, 32, 32, 64, 64, 120, 120, 360, 360, 720 and 720 min. In the whole adsorption experiment, except for part of the time, 20 mL of the MB solution with the corresponding concentration was used, and 10 mg of adsorbent was used for the rest except for part of the dose and the time. Besides the experiment of the temperature part, the rest of the adsorption processes were carried out in the water bath oscillator at 298 K. In order to ensure that the adsorption equilibrium was achieved, the contact time of all adsorption processes was set to 48 h (according to Section 3.2.2 in this paper). The removal effect of the F-C-G beads on the MB was expressed by the removal rate *R_e_* (%). The formula of the removal rate *R_e_* (%) was as follows:

(1) $$R_{e}=\frac{c_{0}-c_{e}}{c_{0}}\times 100\%$$

where *c*_0_ (mg·L^−1^) means the initial concentration of the MB in this study and *c_e_* (mg·L^−1^) is the concentration at adsorption equilibrium.

The adsorbent adsorption capacity is expressed by *q_e_* (mg·g^−1^), which means the amount of MB that can be removed per gram of adsorbent. The equation of the adsorption quantity *q_e_* (mg·g^−1^) of the F-C-G bead when adsorption reaches equilibrium is:

(2) $$q_{e}=\frac{c_{0}-c_{e}}{m}\times V$$

where *V* (L) represents the volume of the adsorbed solution and *m* (g) indicates the amount of adsorbent.

### 2.7. Model Selection Criteria

Several criteria for determining the reliability of the data models were introduced before fitting the experimental data with the adsorption kinetics and isotherms. The Akaike Information Criterion (AIC) is the most widely used criterion [27]. The AIC formula is as follows:

(3) $$\mathrm{AIC}=2p+N\ln\left(\frac{\mathrm{SSE}}{N}\right)$$

When the sample size is small, *N*/*P* < 40, the modified Akaike information criterion (AIC_C_) is generally used to determine the reliability of the model, and its formula is as follows:

(4) $${\mathrm{AIC}}_{C}=\mathrm{AIC}+\left[\frac{2p\left(p+1\right)}{N-p-1}\right]$$

where *p* is the number of parameters in the model, *SSE* is the sum of squares of errors and *N* is the number of data (sample size). The smaller the AIC and AIC_C_ value is, the more reliable the model is [27]. The Akaike weight (*w_i_*) is an important parameter when using the AIC. The *w_i_* calculation formula is as follows [28].

(5) $$w_{i}=\frac{\exp\left(-0.5\Delta {\mathrm{AIC}}_{C\left(i\right)}\right)}{\sum_{i=1}^{R}\exp\left(-0.5\Delta {\mathrm{AIC}}_{C\left(i\right)}\right)}$$

(6) $$\Delta {\mathrm{AIC}}_{C\left(i\right)}={\mathrm{AIC}}_{C\left(i\right)}-{\mathrm{AIC}}_{C,min}$$

where R is the number of models, AIC_C,*min*_ is the minimum AIC_C_ value of all models and AIC_C(*i*)_ is the AIC_C_ value of each model. Assuming that the sum of all *w_i_* is 1, the model with the highest *w_i_* is considered to be the best model.

The Bayesian information criterion (BIC) and the Hannan information criterion (HIC) calculation formulas are as follows [24].

(7) $$\mathrm{BIC}=plnN+N\ln\left(\frac{\mathrm{SSE}}{N}\right)$$

(8) $$\mathrm{HIC}=2pln\left(lnN\right)+N\ln\left(\frac{\mathrm{SSE}}{N}\right)$$

As with the AIC, the smaller the BIC and HIC value is, the more reliable the model is.

The above criteria were used in this study to judge the reliability of the data model. Their results will be analyzed in Section 3.3.

### 2.8. Regeneration Experiments

In this study, the regeneration ability of the F-C-G beads was investigated by the sorption–desorption cycle. After the adsorption equilibrium, the F-C-G beads were desorbed with 0.1 M HCl solution for 8 h to regenerate the F-C-G beads [29]. They were then washed several times with deionized water to remove excess hydrochloric acid. The cleaned adsorbent was freeze-dried in a vacuum freeze dryer, and then placed in the MB solution again for adsorption. Finally, the process was repeated five times.

## 3. Results and Discussions

### 3.1. Characterization Results of Materials

#### 3.1.1. BET Analysis

**Figure 2:**
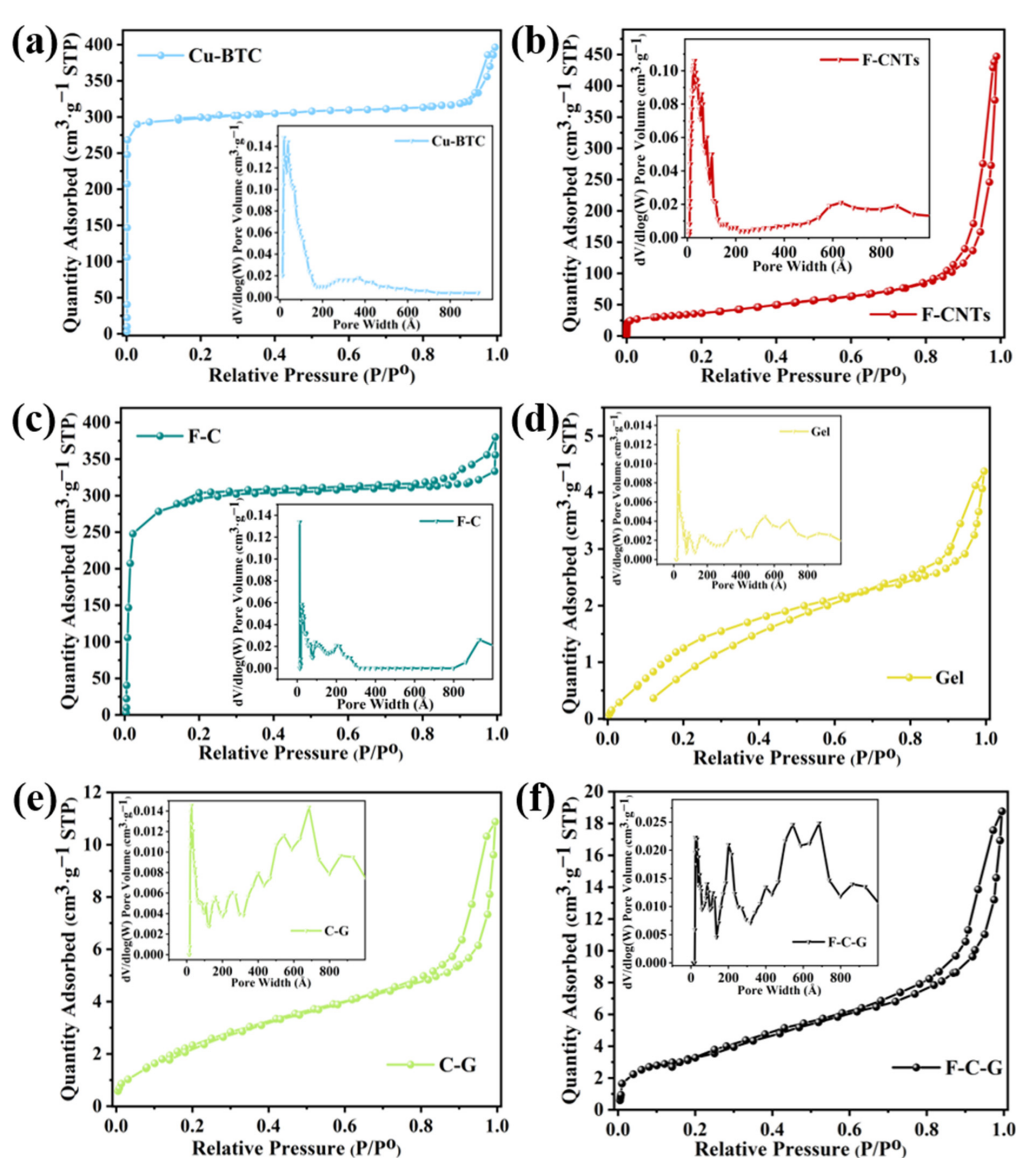
N_2_ adsorption–desorption isotherms and pore size distributions (see insets) of different materials: (**a**) Cu-BTC, (**b**) F-CNTs, (**c**) F-C, (**d**) Gel, (**e**) C-G, (**f**) F-C-G.

Figure 2 demonstrates the BET specific surface area and pore size distribution of the different materials. The N_2_ adsorption curves of Cu-BTC (Figure 2a), F-CNTs (Figure 2b) and F-C (Figure 2c) were II-type isotherms. Due to the strong interaction between the adsorbent and the surface, the adsorption capacity increased rapidly at low relative pressure, and the curve was convex [30]. The specific surface areas of the Cu-BTC, F-CNTs and F-C were 643 ± 22, 131 ± 4 and 618 ± 16 m^2^·g^−1^, respectively. The adsorption curves of the Gel (Figure 2d), C-G (Figure 2e) and F-C-G (Figure 2f) were close to IV-type isotherms, which are similar to type II isotherms, but there was an adsorption hysteresis ring in the middle segment [31]. The specific surface areas of the Gel, C-G and F-C-G were 7.3 ± 0.7, 14.5 ± 0.5 and 19.0 ± 0.3 m^2^·g^−1^, respectively. It can be concluded that the specific surface areas of those different carbon material powders in this study were larger than that of the materials containing polymers. The pore size distribution (see insets in Figure 2) of the three carbon material powders was mainly micropore and mesoporous, while the content of micropore and mesoporous in gelatin was less. The increase of microporous and mesoporous structures can be observed in the C-G composites with Cu-BTC. In the pore size distribution of the F-C-G composite containing F-C, it can be seen that, compared with the C-G composite, microporous and mesoporous structures were further increased. Interestingly, although the specific surface area of the F-C powder was lower than that of the Cu-BTC powder, the specific surface area of F-C-G was larger than that of C-G in the comparison process of them. This is because F-CNTs play the role of the connecting skeleton in the composite material, so that more carbon nanomaterials can be exposed on the surface of the gelatin, and therefore the surface of the adsorbent was rougher. In addition, although the pore size distribution of the material is an important factor affecting the specific surface area, the adsorption capacity depends on the situation. For example, the specific surface area of the carbon nanomaterial in this experiment is much higher than that of the adsorbent containing gelatin, but its adsorption capacity for MB was not very outstanding. On the one hand, it is necessary to consider the content of effective functional groups, and on the other hand, the high specific surface area of carbon nanomaterials is related to the large number of microporous structures. Moreover, if the pore structure is too small, the MB molecules will be difficult to enter, resulting in inadequate use of the contact site.

#### 3.1.2. SEM Analysis

**Figure 3:**
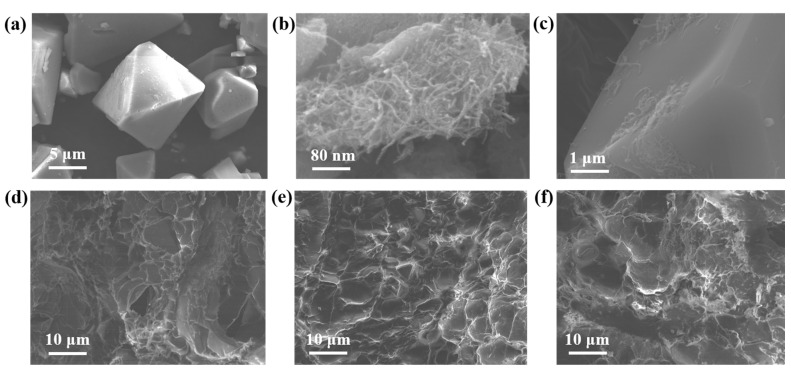
SEM images of different materials: (**a**) Cu-BTC, (**b**) F-CNTs, (**c**) F-C, (**d**) Gel, (**e**) C-G, (**f**) F-C-G.

SEM results of six different materials are demonstrated in Figure 3a–f. Figure 3a indicates the surface topography of Cu-BTC. It is obvious that the Cu-BTC particles prepared in this experiment had a positive octahedral structure, which was similar to previous results [32]. It can be seen from Figure 3b that the F-CNTs had elongated structures. Figure 3c shows the surface topography of F-C, from which it can be seen that the F-CNTs were closely connected with Cu-BTC, indicating that the F-CNTs were successfully grown on the surface of Cu-BTC in this study, so that the specific surface area of the F-C was greater than that of Cu-BTC, and the BET results in Section 3.1.1 also confirm this conclusion. Figure 3d indicates the surface morphology of the Gel, and it can be seen that there were a lot of folds on the surface. C-G beads (Figure 3e) had more folds than the Gel, and Cu-BTC particles coated by gelatin can be seen. The surface morphology of the F-C-G beads (Figure 3f) was similar to that of the C-G beads, and the surface roughness was higher, so it can be inferred that F-C-G had a larger specific surface area. This is consistent with the previous BET analysis in Section 3.1.1.

#### 3.1.3. TGA and DTG Results

**Figure 4:**
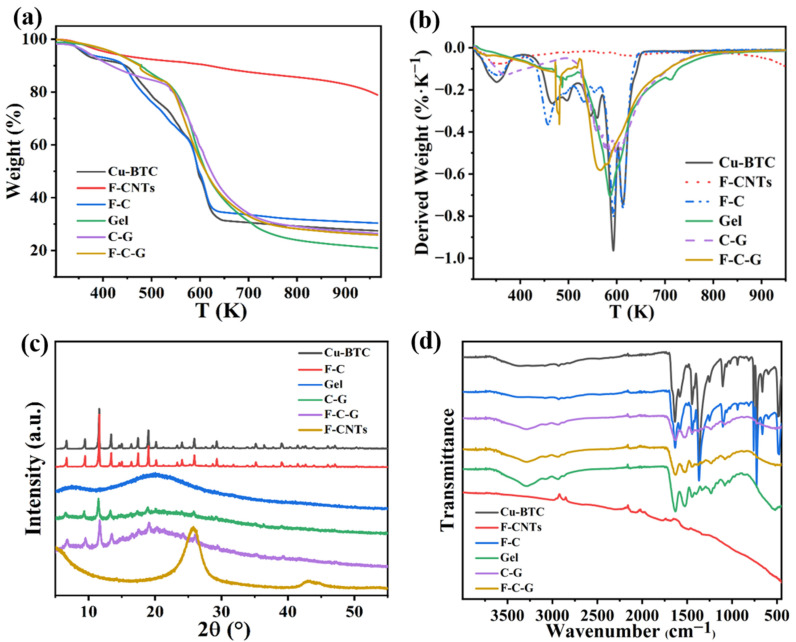
TGA curve (**a**) and DTG curve (**b**) of different materials; XRD (**c**) and FTIR (**d**) results of different materials.

The results of the thermogravimetric analysis (TGA) curves of the six materials are shown in Figure 4a and the derivative thermogravimetry (DTG) curves are demonstrated in Figure 4b. When heated to about 473 K, the weight of the six materials was about 5–10%, which was mainly the weight of the water contained in the materials. In addition, on the DTG curve, there is a peak below 373 K, which is caused by the loss of adsorbed and dislocated water molecules in the material [33]. Approximately 3% of the weight of the F-CNTs was lost between 473 and 673 K, mainly due to the breakdown of the carboxyl groups in the F-CNTs. Cu-BTC lost about 30% of its weight between 473 and 593 K, while there are several weak peaks on the DTG curves, which was related to the loss of free and bound solvent molecules (DMF) [34]. Between 593 and 630 K, Cu-BTC lost about 30% of its weight, and the peak intensity on the DTG curve here is the strongest, which was mainly due to the decomposition of the Cu-BTC ligand structure [35]. Moreover, in this temperature range, there are also strong peaks on the DTG curves of other materials, which indicates that the carboxyl and ester groups in the materials decomposed and became volatiles [36]. After 630K, the weight of the sample hardly changed, and there were no peaks on the DTG curve, indicating that only the carbon structure was left in the sample. The TGA curve of F-C is very similar to that of Cu-BTC, but the final residual weight was about 3% more than that of Cu-BTC, which indicated to some extent that the F-C was successfully synthesized in this experiment. The thermal decomposition process of the Gel was divided into three steps: the first step was to lose 5% water weight before 473 K; the second step was that the sample was pyrolyzed between 473 and 723 K, resulting in about a 60% weight loss; the third step was the carbonization of the sample after 723 K [37]. The TGA curve shape of the C-G and F-C-G is similar to that of the Cu-BTC and Gel, which proved the successful synthesis of the gelatin matrix composites in this experiment. The difference of the maximum thermal degradation temperature of the different materials can be analyzed by comparing the rightmost peak of the different DTG curves. Obviously, the maximum thermal degradation temperatures of the six materials are around 600 K. However, the peak of the F-C-G shifted to the left, indicating that F-C-G requires less energy to release volatiles than other materials [36].

#### 3.1.4. XRD Results

The XRD spectra of the six materials are shown in Figure 4c. The graph of the pure Cu-BTC had a specific phase peak, which was almost consistent with the previous literature data [32]. The graph of the pure F-CNTs was similar to the data in previous studies [38], with a peak near 26° and a low peak near 43°. The phase peak shape of the F-C XRD image was almost the same as that of the Cu-BTC, indicating that the F-C composite material had been successfully synthesized in this experiment. Therefore, the addition of the F-CNTs did not affect the coordination of the Cu-BTC during the synthesis of the F-C. Pure gelatin is a polymer, so there was almost no phase peak in its XRD image, and the image shape was similar to the previous research data [39]. The positions of the phase peaks in the XRD images of C-G and F-C-G were the same as those of Cu-BTC and F-C, and the overall graphs were convex near 20°, which were similar to the graph of the Gel. The XRD results showed that the C-G and F-C-G composite materials in this experiment were successfully prepared.

#### 3.1.5. FTIR Results

As shown in Figure 4d, functional groups contained in the six materials were characterized by the FTIR spectrum. The wide peak at 3280 cm^−1^ represents the associated -OH in the material. Except for the curve of the F-CNTs, the wide peak can be seen in the curves of the other five materials, which indicates that the surface of the F-CNTs prepared in this experiment contains no or only a very small amount of hydroxyl. The peaks at 2890 cm^−1^ and 1716 cm^−1^ represent -OH and C=O on the carboxyl functional groups. There are peaks here on the curves of all the six materials, indicating that they all contain carboxyl functional groups. The peak at 1620 cm^−1^ is N-H in the amide group, which proves the presence of Gel in the material containing Gel. The peaks at 1587 cm^−1^ and 725 cm^−1^ are C=C on the benzene ring and C-H on the disubstituted benzene ring, indicating that the above materials contain a benzene ring structure. The FTIR spectrum results are similar to previous studies [40]. It can be seen from the FTIR spectrum results that the peak strength of the Cu-BTC and F-C powders on the curve are weakened on the whole after the composite with the Gel, which may be due to the hydrogen bond force formed by the two different materials after the composite weakens the strength of the above peaks.

### 3.2. Results of Adsorption Experiments

#### 3.2.1. Comparison of Adsorption Properties of Six Adsorbents

**Figure 5:**
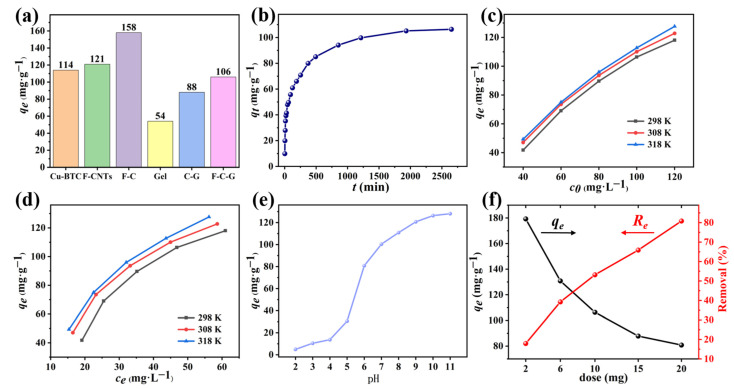
Comparison of adsorption capacity of different materials (**a**); Effects of time (**b**), initial concentration (**c**), temperature (**d**), pH (**e**), dose (**f**) on the adsorption process.

Figure 5a shows the equilibrium adsorption capacity of Cu-BTC, F-CNTs, F-C, Gel, C-G and F-C-G to MB, which were 114, 121, 158, 54, 88 and 106 mg·g^−1^, respectively, and the standard deviation (σ) of the maximum adsorption capacity of each material were 2, 3, 1.7, 2.2, 2.8 and 2.6. Apparently, after the combination of the Cu-BTC and F-CNTs to obtain F-C, the adsorption capacity of the F-C was higher than its two parent materials, which was similar to the results of V. Jabbari et al. studies [20]. As can be seen from the above results of the adsorption capacity of different materials, the Gel had the worst adsorption capacity due to its smallest specific surface area. The adsorption capacities of the C-G and F-C-G after composite with the Gel were better than that of the Gel but worse than that of the three carbon materials powder. This was because the Gel covered most of the carbon powder particles, so that the specific surface area of the composite was smaller than that of the carbon powder.

#### 3.2.2. Effects of Different Conditions

The influence of the contact time on the adsorption effect is illustrated in Figure 5b. At the early stage of the adsorption process, the removal rate of the MB was faster because there were a large number of adsorption sites on the surface of the material for the attachment of MB molecules [41]. With the increase of time, the rate of adsorption gradually slowed down and reached equilibrium at about 2000 min. This was because, during the adsorption process, the MB molecules were adsorbed on the F-C-G bead surface, and the adsorption site was gradually occupied. In addition, with the progress of the adsorption process, the concentration of MB in the solution also decreased, and the reduction of the driving force of the concentration gradient would also lead to the decrease of the adsorption rate [42].

The influence of the initial concentration of MB on the adsorption process of the adsorbent is shown in Figure 5c. At a lower initial concentration (*c*_0_), the equilibrium adsorption capacity (*q_e_*) was small; meanwhile, the *q_e_* got bigger with the increase of the *c*_0_. This is because the higher the concentration of MB, the greater the concentration gradient driving force, the more fully utilized the adsorption sites on the surface of the adsorbent. However, as *c*_0_ increased, the rate of increase in *q_e_* decreased. The reason for this phenomenon was that the adsorption site was gradually occupied and the influence of *c*_0_ on *q_e_* was gradually weakened [43].

As shown in Figure 5d, different temperatures exert influence on the adsorption process. According to the experimental data, there were differences in the adsorption capacity at different temperatures (*q_e_*_,318K_ > *q_e_*_,308K_ > *q_e_*_,298K_). That the adsorption effect became better because of the higher temperature implied that the adsorption behavior of the F-C-G beads on MB was endothermic [18].

The initial pH is an essential factor influencing the adsorption performance of the dyes. The degree of ionization of the dye and the surface charge of the adsorbent will change with the pH value [44].The results of this study were similar to the previous study [45]. The pH range set in this experiment was 2–11. The influence of the pH on the adsorption process of the F-C-G for MB is demonstrated in Figure 5e. The adsorption amount of MB was small at a low pH value, and then increased with the increase of the pH. Nonetheless, when the pH > 9, the *q_e_* almost did not increase with the increase of the pH. When in acidic solution, excessive H^+^ ions on the surface of the F-C-G compete with the MB molecules, and there would be electrostatic repulsion between the positively charged and cationic dye MB molecules on the surface of adsorbent, resulting in reduced adsorption capacity [46]. When in an alkaline solution, H^+^ ions on the surface of the F-C-G would dissociate into an aqueous solution, making the surface of the material negatively charged, thus enhancing the adsorption capacity under the action of electrostatic attraction. In addition, the natural pH of 100 mg·L^−1^ MB solution measured in this experiment was 7.89. In summary, the adsorption effect of the F-C-G on MB was better under alkaline conditions, and because the natural pH of the MB solution itself was alkaline, the natural pH of the MB solution also had a good adsorption effect [40].

The influence of the adsorbent dose on the adsorption effect is illustrated in Figure 5f. The removal rate of MB increased from 18% to 81% and the adsorption capacity decreased from 179 mg·g^−1^ to 81 mg·g^−1^ (σ = 3.2, 3.3, 2.6, 2.8, 1.7) when different doses of the F-C-G beads were added to the MB solution. This is attributed to the increase of the F-C-G beads, as more adsorption sites were afforded, thus increasing the removal rate of the MB. As the adsorbent increased, the utilization rate of the adsorption sites on the F-C-G beads decreased, resulting in a decrease in the adsorption capacity of the F-C-G adsorbents.

### 3.3. Adsorption Kinetics and Adsorption Isotherm Analysis

#### 3.3.1. Adsorption Kinetics

**Figure 6:**
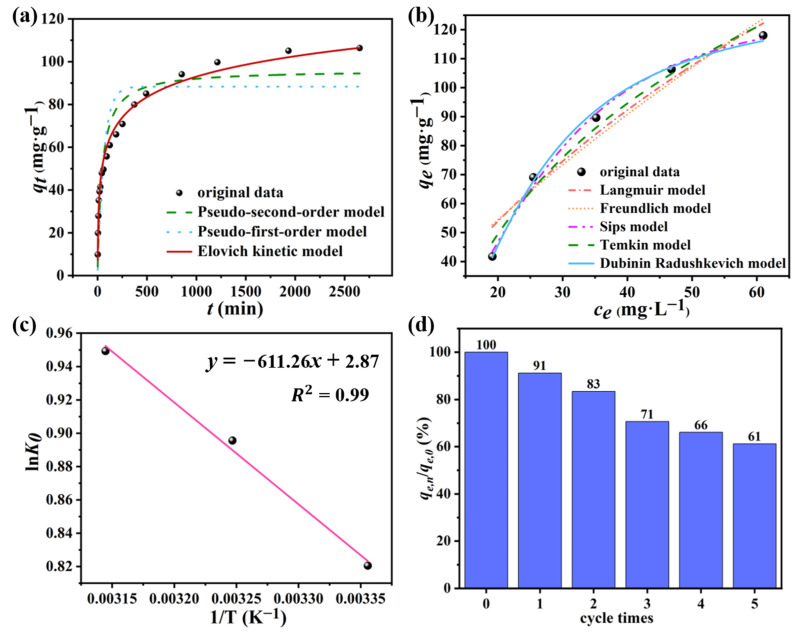
Adsorption kinetic model fitting (**a**); Adsorption isotherm fitting (**b**); Fitting results of thermodynamic calculation (**c**); Regeneration performance (**d**).

Pseudo-first-order (PFO), pseudo-second-order (PSO) and Elovich models were used in this paper to fit the data of the influence of time on the adsorption process, and the curve of the fitting results is demonstrated in Figure 6a. The three adsorption kinetics models all illustrated that the adsorption rate was fast in the early stage (about 250 min before), because a mass of adsorption sites on the surface of the adsorbent were not utilized so that the MB molecules were quickly captured by the adsorbent. Moreover, as time goes on, the adsorption rate gradually decreased. This is because, as the adsorption process progressed, the adsorption sites on the surface of the composite were gradually occupied by the MB molecules that had been captured, and took a while for the MB molecules to diffuse into the F-C-G composite [29]. Until the late stage of the adsorption, the increase of the adsorption capacity (*q_t_*) was not obvious, which is because the adsorption sites in the composite were almost all occupied, reaching adsorption saturation. Therefore, it can be considered that the adsorption process of MB by the new composite reached equilibrium after 2000 min. The PFO model can be considered as a physical adsorption model to describe the adsorption process. The PSO model can be regarded as a chemisorption-dominated model [47]. The Elovich model describes the adsorption behavior of pollutants on the surface of heterogeneous solid adsorbent [48]. The formulas of the three dynamic models are as follows:

(9) $$\mathrm{PFO}\;\mathrm{model}:\;q_{t}=q_{e}\left(1-e^{-k_{1}t}\right)$$

(10) $$\mathrm{PSO}\;\mathrm{model}:\;q_{t}=\frac{k_{2}q_{e}^{2}t}{1+k_{2}q_{e}t}$$

(11) $$\mathrm{Elovich}\;\mathrm{kinetic}\;\mathrm{model}:\;q_{t}=\frac{\ln\left(\alpha \beta \right)}{\beta }+\frac{\ln\left(t\right)}{\beta }$$

where *q_t_* (mg·g^−1^) refers to the time-dependent amount of MB adsorption by the F-C-G beads per unit mass, *t* (min) is the contact time, *q_e_* (mg·g^−1^) stands for the equilibrium adsorption amount, *k*_1_ (min^−1^) and *k*_2_ (min^−1^) means the rate constant of the PFO and PSO models and α (mg·g^−1^·min^−1^) and β (g·mg^−1^) are the initial adsorption rates and the parameters related to chemisorption activation energy and surface coverage extent, respectively.

The values of the parameters in the formulas of the three adsorption kinetics models and the reliability evaluation results of the models are demonstrated in Table 1. Firstly, the fitting degree of the models was preliminarily judged by the value of R^2^. The fitting degree of the Elovich model (R^2^ = 0.99) was higher than that of the PFO model (R^2^ = 0.79) and PSO model (R^2^ = 0.89). Therefore, it can be preliminarily determined that the data of the influence of time on the adsorption behavior in this experiment were more consistent with the Elovich model. Then, several information criteria were used to evaluate the reliability of the three adsorption kinetics models. The results in Table 1 show that the values of the AIC, AIC_C_, BIC and HIC of the Elovich model are the smallest among the three models, while the values of *w_i_* are the largest. Therefore, the Elovich model has the highest reliability. Thus, it can be concluded that the adsorption behavior of the F-C-G adsorbent on MB can be described by the Elovich model, indicating that the surface of the F-C-G composite was a heterogeneous solid surface.

#### 3.3.2. Adsorption isotherm

Langmuir, Freundlich, Sips, Temkin and Dubinin Radushkevich (D-R) isotherm models were used in this paper to fit the data of this study at 298 K, and the curve of fitting results are shown in Figure 6b. The Langmuir model is commonly used to describe the adsorption on a single uniform adsorbent surface [49]. The Freundlich model shows that the adsorption process occurs on multilayer heterogeneous surfaces [50]. The Sips isotherm model is a formula that improved from the Langmuir and Freundlich models, and the closer *n* value in the formula is to 1, the more uniform adsorbent surface is [13]. The Temkin model is an isotherm model used to describe the relationship between adsorbent and adsorption heat [51]. The D-R model is used to distinguish whether the adsorption process is related to physical adsorption, ion exchange or chemical adsorption [48]. The formula of the five isotherm models is as follows:

(12) $$\mathrm{Langmuir}:\;q_{e}=\frac{q_{m}K_{L}c_{e}}{1+K_{L}c_{e}}$$

(13) $$\mathrm{Freundlich}:\;q_{e}=K_{F}c_{e}^{1/n}$$

(14) $$\mathrm{Sips}:\;q_{e}=\frac{q_{m}{\left(K_{S}c_{e}\right)}^{b}}{1+{\left(K_{S}c_{e}\right)}^{b}}$$

(15) $$\mathrm{Temkin}:\;q_{e}=B_{T}\ln\left(A_{T}C_{e}\right)$$

(16) $$D-R:\;q_{e}=q_{m}\exp[-A_{D}\left(\ln\left(1+\frac{1}{C_{e}}\right){)}^{2}\right]$$

where *c_e_* (mg·L^−1^) represents the concentration of the MB solution at equilibrium after adsorption; *q_m_* (mg·g^−1^) means the theoretical maximum adsorption capacity obtained by model fitting; *q_e_* (mg·g-1) is the equilibrium adsorption capacity measured in practice; *K_L_* (L·mg^−1^) represents the parameters in the Langmuir isotherm model, which is related to adsorption energy; *K_F_* (mg^1−1/n^·L^1/n^·g^−1^) represents the parameter in the Freundlich isotherm model, which is related to adsorption capacity; *n* represents the parameters related to adsorption strength; *K_S_* [(L/mg)^1/b^] represents the parameters in the Sips model; *b* (dimensionless) means the heterogeneity constant of Sips; *B_T_* (J·mol^−1^) describes the energy constant in the Temkin isotherm model, while *A_T_* (L·mg^−1^) represents the equilibrium binding constant; *A_D_* = *K_D_R*^2^*T*^2^, where *K_D_* (mol^2^·J^−2^) describes the parameters in the D–R model which is related to the adsorption energy; *R* means the universal gas constant (8.314 J·mol^−1^·K^−1^); *E* represents the average free energy in the adsorption process, and its calculation formula is as follows:

(17) $$E=\frac{1}{\sqrt{2K_{D}}}$$

By calculating the value of *E*, the type of adsorption process of the F-C-G beads on MB can be determined. If *E* < 8 kJ·mol^−1^, it can be considered that the physical adsorption is dominant in the adsorption process. If 8 kJ·mol^−1^ < *E* < 16 kJ·mol^−1^, it can be inferred that the adsorption process is ion exchange adsorption. When *E* > 16 kJ·mol^−1^, chemisorption is dominant.

The values of each parameter of the five adsorption isotherm models and the results of the model reliability evaluation are shown in Table 2. Firstly, the results were preliminarily judged by the value of R^2^. The data in this experiment was more consistent with the Sips and D-R models (R^2^ values of both was 0.99). Then, after the judgment of several information criteria, it is found that the values of AIC, AIC_C_, BIC and HIC of the D-R model are the smallest and *w_i_* is the largest among all isotherm models. Therefore, it can be judged that the adsorption data in this experiment is more consistent with the D-R model. The calculated value of *E* is 83.33 kJ·mol^−1^ (>16 kJ·mol^−1^). To sum up, the adsorption process of F-C-G to MB is a chemisorption-dominated process.

### 3.4. Adsorption Thermodynamic

Because the temperature will affect the movement of the adsorbed molecules in the adsorption process, the energy-dependent mechanism must appear in the whole adsorption process. According to the influence of temperature on the adsorption behavior in Section 3.2.2 of this paper, it can be seen that the adsorption capacity of F-C-G on MB increased with the increase of temperature. To further illustrate the effect of temperature on the adsorption process, thermodynamic analysis of adsorption was carried out by calculating the change of enthalpy change (Δ*H*^0^), entropy change (Δ*S*^0^) and Gibbs free energy change (Δ*G*^0^). The calculation formulas are as follows [47]:

(18) $$\Delta G^{0}=-RTLnK_{0}$$

(19) $$\ln K_{0}=-\frac{\Delta H^{0}}{RT}+\frac{\Delta S^{0}}{R}$$

(20) $$K_{0}=\frac{q_{e}}{c_{e}}$$

where *R* represents the universal gas constant (8.314 J·mol^−1^·K^−1^); Δ*G* (kJ·mol^−1^), Δ*H*^0^ (kJ·mol^−1^) and *ΔS*^0^ (J·mol^−1^·K^−1^) are Gibbs free energy change, enthalpy change and entropy change, respectively; *T* is the temperature in Kelvin (K); *q_e_* (mg·g^−1^) and *c_e_* (mg·L^−1^) are the adsorption capacity and equilibrium concentration when the adsorption process reaches equilibrium [52].

The corresponding *q_e_* and *c_e_* results (initial concentration is 100 mg·L^−1^) at 303 K, 313 K and 323 K were selected as the basis for the *K*_0_ (L·g^−1^) calculation. The calculated results of *K*_0_ were 2.27 (303 K), 2.45 (313 K) and 2.58 (323 K). By linear fitting *lnK*_0_ with 1/*T*, the values of Δ*H*^0^ and Δ*S*^0^ can be obtained, and then the Δ*G* can be calculated (see Figure 6c). The results are listed in Table 3. The Gibbs free energy changes (Δ*G*) of the adsorption of MB by the F-C-G beads were −2.03 (298 K), −2.27 (308 K) and −2.51 (318 K) kJ·mol^−1^ at different temperatures, respectively. The adsorption process of MB by the F-C-G beads was spontaneous because of Δ*G* < 0. Additionally, with the increase of temperature, the absolute value of Δ*G* went up, indicating that the adsorption process became more favorable with the increase of temperature. Moreover, Δ*H* > 0 indicates that the adsorption process of the F-C-G beads on MB was endothermic, and Δ*S* > 0 indicated that the disorder and randomness at the solid–solution interface increased after the adsorption of MB by the F-C-G [53].

### 3.5. Results of Regeneration Experiment

The regeneration capacity of the F-C-G beads was evaluated by the ratio between the adsorption capacity of the adsorbent after the regeneration for n times (*q_e, n_*) and that of the adsorbent without regeneration (*q_e,_*_0_). The measured results are shown in Figure 6d. The results showed that the adsorption effect of the F-C-G on MB gradually weakened with the increase of regeneration times. After five cycles of adsorption–desorption, the adsorption effect of the F-C-G beads on MB was 61.23% compared with that of the adsorbent without the regeneration treatment.

### 3.6. Analysis of Adsorption Mechanism

**Figure 7:**
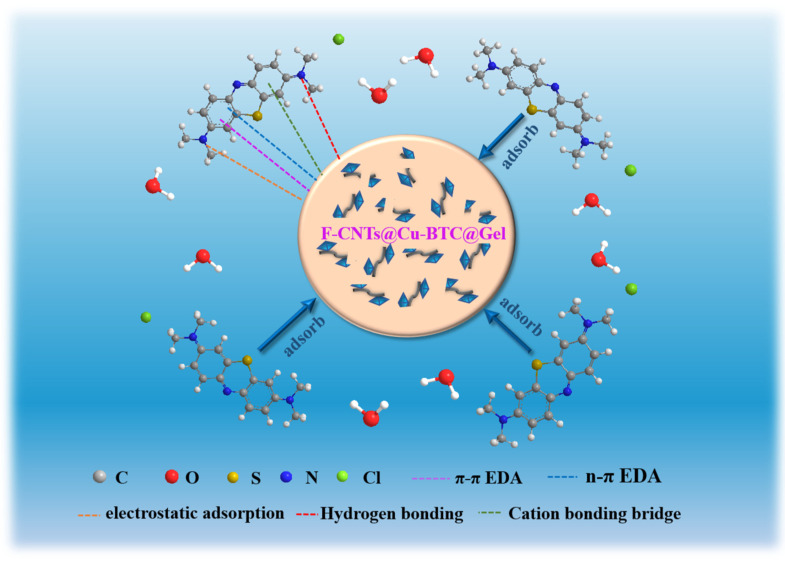
Adsorption mechanism of F-C-G for MB.

Figure 7 shows the adsorption mechanism of the F-C-G beads for MB. Firstly, MB is a cationic dye, so its molecules in the solution are positively electric [54]. Therefore, there was electrostatic adsorption between the MB molecules and the negative hydroxyl (-OH), amino (-NH_2_) and carboxyl (-COOH) groups on the F-C-G [45]. Secondly, the π-π electron donor–acceptor (π-π EDA) interaction existed between the benzene ring structure on the MB molecules and the Cu-BTC and F-CNTs in the F-C-G. Thirdly, the metal cation bonding bridge existed between the MB molecules and the open Cu^2+^ in Cu-BTC ligand [55]. Finally, there was hydrogen bonding between the MB molecules and the F-C-G, and van der Waals forces must exist between smaller MB molecules and the adsorbent surface [56]. Moreover, the adsorption of MB on F-C-G may also have n-π EDA interaction. The full name of n-π EDA is n-π electron donor–acceptor. Here, n is the electron donor, which can be oxygen atom or nitrogen atom. The aromatic ring on the molecular structure of MB acts as the π electron acceptor, and the carbonyl oxygen (=O) on the F-C-G adsorbent acts as the electron donor, thus forming the n-π EDA interaction between the adsorbent and MB [57].

### 3.7. Comparison of Adsorption of Methylene Blue on Various Adsorbents

The comparison results between other adsorbents previously studied for MB adsorption and the new adsorbents studied in this paper are shown in the Table 4.

## 4. Conclusions

In conclusion, this study prepared a kind of composite bead with good adsorption performance for methylene blue (MB). Firstly, Cu-BTC was grown on the surface of F-CNTs by the hydrothermal method, and F-CNTs@Cu-BTC (F-C) was obtained. Then, F-C particles were coated by gelatin to avoid secondary pollution, so the F-C-G beads were obtained. At 298 K and the initial concentration of 100 mg·L^−1^, the adsorption capacity of the F-C-G beads for MB was 106.5 mg·g^−1^. The adsorption behavior of the F-C-G adsorbent on MB can be described by the Elovich model, indicating that the surface of the F-C-G composite was a heterogeneous solid surface. After the adsorption isotherm analysis, the adsorption process was more consistent with the Dubinin Radushkevich (D-R) model, so the adsorption process of the F-C-G to MB was a chemisorption-dominated process. The adsorption of MB by the F-C-G beads was spontaneous and endothermic. After five regeneration cycles, the adsorption capacity of the F-C-G beads was 61.23% of the original. The F-C-G bead was a new type of composite material with a simple manufacturing process and environmental friendliness, so it may have a good practical application prospect.

## Figures and Tables

**Table 1:** Results of adsorption kinetics model.

Models	Parameters							
Pseudo-first-order model	*k*_1_ (min^−1^)	*q_e_* (mg·g^−1^)	R^2^	AIC	AIC_C_	*w_i_*	BIC	HIC
	1.58 × 10^−2^	88.3	0.79	96.7	97.5	1.79 × 10^−12^	98.5	97.0
Pseudo-second-order model	*k*_2_ (min^−1^)	*q_e_* (mg·g^−1^)	R^2^	AIC	AIC_C_	*w_i_*	BIC	HIC
	2.35 × 10^−4^	96.1	0.89	83.7	84.5	1.20 × 10^−9^	85.5	84.0
Elovich kinetic model	*α* (mg·g^−1^·min^−1^)	*β* (g·mg^−1^)	R^2^	AIC	AIC_C_	*w_i_*	BIC	HIC
	11.7	0.07	0.99	42.6	43.4	0.99	44.4	42.9

**Table 2:** Results of the adsorption isotherm model.

Models	Parameters								
Langmuir	*q_m_* (mg·g^−1^)	*K_L_* (L·mg^−1^)		R^2^	AIC	AICc	*w_i_*	BIC	HIC
	320	0.01		0.94	21.8	27.8	1.41 × 10^−3^	21.0	19.7
Freundlich	*K_f_* (mg^1−1/n^·L^1/n^·g^−1^)	*n*		R^2^	AIC	AICc	*w_i_*	BIC	HIC
	5.97	1.36		0.93	23.3	29.3	6.50 × 10^−4^	22.6	21.3
Sips	*q_m_* (mg·g^−1^)	*K_S_* [(L·mg^−1^)^1/b^]	b	R^2^	AIC	AICc	*w_i_*	BIC	HIC
	129	0.04	2.55	0.99	11.6	35.6	2.82 × 10^−5^	10.5	8.47
Temkin	*B_T_* (J·mol^−1^)	*A_T_* (L·mg^−1^)		R^2^	AIC	AICc	*w_i_*	BIC	HIC
	65.0	0.11		0.98	17.1	23.1	1.50 × 10^−2^	16.3	15.0
D-R	*q_m_* (mg·g^−1^)	A_D_	E (kJ·mol^−1^)	R^2^	AIC	AICc	*w_i_*	BIC	HIC
	131	442	83.3	0.99	8.70	14.7	0.98	7.92	6.60

**Table 3:** Results of thermodynamic analysis.

Δ*H*^0^ (kJ·mol^−1^)	Δ*S*^0^ (J·mol^−1^·K^−1^)	Δ*G*^0^ (kJ·mol^−1^)		
		298 K	308 K	318 K
5.08	23.9	−2.03	−2.27	−2.51

**Table 4:** Comparison of MB with different adsorbents.

Adsorbent	Experimental Conditions			Ref.
	*T* (K)	*c*_0_ (mg·L^−1^)	*q_e_* (mg·g^−1^)	
CuC21	N/A	100	172	[20]
Cu-BTC	298	100	39.5	[14]
GTP	N/A	469	40.1	[40]
Gelatin hydrogel	N/A	100	60.3	[26]
Gelatin-CNT-MNPs	N/A	1000	466	[18]
F-C	298	100	158	This work
C-G	298	100	88.2	This work
F-C-G	298	100	106	This work

## Data Availability

The data presented in this study are available on request from the corresponding authors.

## References

[1] Youcef L.D., Belaroui L.S., Lopez-Galindo A. (2019). Adsorption of a cationic methylene blue dye on an Algerian palygorskite. Appl. Clay Sci..

[2] Alver E., Metin A.U., Brouers F. (2020). Methylene blue adsorption on magnetic alginate/rice husk bio-composite. Int. J. Biol. Macromol..

[3] Nguyen C.H., Fu C.-C., Juang R.-S. (2018). Degradation of methylene blue and methyl orange by palladium-doped TiO_2_ photocatalysis for water reuse: Efficiency and degradation pathways. J. Clean. Prod..

[4] Zhang Y., Li Y., Xu W., Cui M., Wang M., Chen B., Sun Y., Chen K., Li L., Du Q. (2022). Filtration and adsorption of tetracycline in aqueous solution by copper alginate-carbon nanotubes membrane which has the muscle-skeleton structure. Chem. Eng. Res. Des..

[5] Lyu H., Gao B., He F., Zimmerman A.R., Ding C., Tang J., Crittenden J.C. (2018). Experimental and modeling investigations of ball-milled biochar for the removal of aqueous methylene blue. Chem. Eng. J..

[6] Machado T.F., Serra M.E.S., Murtinho D., Valente A.J.M., Naushad M. (2021). Covalent Organic Frameworks: Synthesis, Properties and Applications—An Overview. Polymers.

[7] Munir M., Nazar M.F., Zafar M.N., Zubair M., Ashfaq M., Hosseini-Bandegharaei A., Khan S.U.-D., Ahmad A. (2020). Effective Adsorptive Removal of Methylene Blue from Water by Didodecyldimethylammonium Bromide-Modified Brown Clay. ACS Omega.

[8] Yang Y., Zhu Q., Peng X., Sun J., Li C., Zhang X., Zhang H., Chen J., Zhou X., Zeng H. (2022). Hydrogels for the removal of the methylene blue dye from wastewater: A review. Environ. Chem. Lett..

[9] Kumar A., Jena H.M. (2016). Removal of methylene blue and phenol onto prepared activated carbon from Fox nutshell by chemical activation in batch and fixed-bed column. J. Clean. Prod..

[10] Soni S., Bajpai P., Mittal J., Arora C. (2020). Utilisation of cobalt doped Iron based MOF for enhanced removal and recovery of methylene blue dye from waste water. J. Mol. Liq..

[11] Li Y., Du Q., Liu T., Peng X., Wang J., Sun J., Wang Y., Wu S., Wang Z., Xia Y. (2013). Comparative study of methylene blue dye adsorption onto activated carbon, graphene oxide, and carbon nanotubes. Chem. Eng. Res. Des..

[12] Li Y.-Z., Wang G.-D., Yang H.-Y., Hou L., Wang Y.-Y., Zhu Z. (2020). Novel cage-like MOF for gas separation, CO_2_ conversion and selective adsorption of an organic dye. Inorg. Chem. Front..

[13] Chen B., Li Y., Li M., Cui M., Xu W., Li L., Sun Y., Wang M., Zhang Y., Chen K. (2021). Rapid adsorption of tetracycline in aqueous solution by using MOF-525/graphene oxide composite. Microporous Mesoporous Mater..

[14] Li Y., Gao C., Jiao J., Cui J., Li Z., Song Q. (2021). Selective Adsorption of Metal–Organic Framework toward Methylene Blue: Behavior and Mechanism. ACS Omega.

[15] Lin S., Song Z., Che G., Ren A., Li P., Liu C., Zhang J. (2014). Adsorption behavior of metal–organic frameworks for methylene blue from aqueous solution. Microporous Mesoporous Mater..

[16] Lahtinen E., Precker R.L.M., Lahtinen M., Hey-Hawkins E., Haukka M. (2019). Selective Laser Sintering of Metal-Organic Frameworks: Production of Highly Porous Filters by 3D Printing onto a Polymeric Matrix. ChemPlusChem.

[17] Pei R., Fan L., Zhao F., Xiao J., Yang Y., Lai A., Zhou S.-F., Zhan G. (2020). 3D-Printed metal-organic frameworks within biocompatible polymers as excellent adsorbents for organic dyes removal. J. Hazard. Mater..

[18] Saber-Samandari S., Saber-Samandari S., Joneidi-Yekta H., Mohseni M. (2017). Adsorption of anionic and cationic dyes from aqueous solution using gelatin-based magnetic nanocomposite beads comprising carboxylic acid functionalized carbon nanotube. Chem. Eng. J..

[19] Zhang Y., Wibowo H., Zhong L., Horttanainen M., Wang Z., Yu C., Yan M. (2021). Cu-BTC-based composite adsorbents for selective adsorption of CO_2_ from syngas. Sep. Purif. Technol..

[20] Jabbari V., Veleta J., Zarei-Chaleshtori M., Gardea-Torresdey J., Villagrán D. (2016). Green synthesis of magnetic MOF@GO and MOF@CNT hybrid nanocomposites with high adsorption capacity towards organic pollutants. Chem. Eng. J..

[21] Rigueto C.V.T., Rosseto M., Nazari M.T., Ostwald B.E.P., Alessandretti I., Manera C., Piccin J.S., Dettmer A. (2021). Adsorption of diclofenac sodium by composite beads prepared from tannery wastes-derived gelatin and carbon nanotubes. J. Environ. Chem. Eng..

[22] Santoso E., Ediati R., Kusumawati Y., Bahruji H., Sulistiono D.O., Prasetyoko D. (2020). Review on recent advances of carbon based adsorbent for methylene blue removal from waste water. Mater. Today Chem..

[23] Zhang X., Li Y., Wu M., Pang Y., Hao Z., Hu M., Qiu R., Chen Z. (2021). Enhanced adsorption of tetracycline by an iron and manganese oxides loaded biochar: Kinetics, mechanism and column adsorption. Bioresour. Technol..

[24] Cao Y., Dong S., Dai Z., Zhu L., Xiao T., Zhang X., Yin S., Soltanian M.R. (2021). Adsorption model identification for chromium (VI) transport in unconsolidated sediments. J. Hydrol..

[25] Li Y., Liu F., Xia B., Du Q., Zhang P., Wang D., Wang Z., Xia Y. (2010). Removal of copper from aqueous solution by carbon nanotube/calcium alginate composites. J. Hazard. Mater..

[26] Ren J., Wang X., Zhao L., Li M., Yang W. (2021). Double Network Gelatin/Chitosan Hydrogel Effective Removal of Dyes from Aqueous Solutions. J. Polym. Environ..

[27] El-Naas M.H., Alhaija M.A., Al-Zuhair S. (2017). Evaluation of an activated carbon packed bed for the adsorption of phenols from petroleum refinery wastewater. Environ. Sci. Pollut. Res..

[28] Bouabidi Z.B., El-Naas M.H., Cortes D., McKay G. (2018). Steel-Making dust as a potential adsorbent for the removal of lead (II) from an aqueous solution. Chem. Eng. J..

[29] Wang M., Li Y., Cui M., Li M., Xu W., Li L., Sun Y., Chen B., Chen K., Zhang Y. (2022). Barium alginate as a skeleton coating graphene oxide and bentonite-derived composites: Excellent adsorbent based on predictive design for the enhanced adsorption of methylene blue. J. Colloid Interface Sci..

[30] Wang W., Tian G., Zhang Z., Wang A. (2015). A simple hydrothermal approach to modify palygorskite for high-efficient adsorption of Methylene blue and Cu(II) ions. Chem. Eng. J..

[31] Yan L., Liu Y., Zhang Y., Liu S., Wang C., Chen W., Liu C., Chen Z., Zhang Y. (2020). ZnCl2 modified biochar derived from aerobic granular sludge for developed microporosity and enhanced adsorption to tetracycline. Bioresour. Technol..

[32] Liu Y., Ghimire P., Jaroniec M. (2019). Copper benzene-1,3,5-tricarboxylate (Cu-BTC) metal-organic framework (MOF) and porous carbon composites as efficient carbon dioxide adsorbents. J. Colloid Interface Sci..

[33] Lucena F.R.S., de Araújo L.C.C., Rodrigues M.d.D., da Silva T.G., Pereira V.R.A., Militão G.C.G., Fontes D.A.F., Rolim-Neto P.J., da Silva F.F., Nascimento S.C. (2013). Induction of cancer cell death by apoptosis and slow release of 5-fluoracil from metal-organic frameworks Cu-BTC. Biomed. Pharmacother..

[34] Li Y., Miao J., Sun X., Xiao J., Li Y., Wang H., Xia Q., Li Z. (2016). Mechanochemical synthesis of Cu-BTC@GO with enhanced water stability and toluene adsorption capacity. Chem. Eng. J..

[35] Sun X., Li H., Li Y., Xu F., Xiao J., Xia Q., Li Y., Li Z. (2015). A novel mechanochemical method for reconstructing the moisture-degraded HKUST-1. Chem. Commun..

[36] Domán A., Klébert S., Madarász J., Sáfrán G., Wang Y., László K. (2020). Graphene Oxide Protected Copper Benzene-1,3,5-Tricarboxylate for Clean Energy Gas Adsorption. Nanomaterials.

[37] Cui L., Xiong Z., Guo Y., Liu Y., Zhao J., Zhang C., Zhu P. (2015). Fabrication of interpenetrating polymer network chitosan/gelatin porous materials and study on dye adsorption properties. Carbohydr. Polym..

[38] Li S., Gong Y., Yang Y., He C., Hu L., Zhu L., Sun L., Shu D. (2015). Recyclable CNTs/Fe_3_O_4_ magnetic nanocomposites as adsorbents to remove bisphenol A from water and their regeneration. Chem. Eng. J..

[39] Alinejad-Mir A., Amooey A.A., Ghasemi S. (2018). Adsorption of direct yellow 12 from aqueous solutions by an iron oxide-gelatin nanoadsorbent; kinetic, isotherm and mechanism analysis. J. Clean. Prod..

[40] Jiang J., Zhang Q., Zhan X., Chen F. (2019). A multifunctional gelatin-based aerogel with superior pollutants adsorption, oil/water separation and photocatalytic properties. Chem. Eng. J..

[41] Arora C., Soni S., Sahu S., Mittal J., Kumar P., Bajpai P.K. (2019). Iron based metal organic framework for efficient removal of methylene blue dye from industrial waste. J. Mol. Liq..

[42] Tang K., Li Y., Zhang X., Li M., Du Q., Li H., Wang Y., Wang D., Wang C., Sui K. (2019). Synthesis of citric acid modified β-cyclodextrin/activated carbon hybrid composite and their adsorption properties toward methylene blue. J. Appl. Polym. Sci..

[43] Dai H., Huang Y., Huang H. (2018). Eco-friendly polyvinyl alcohol/carboxymethyl cellulose hydrogels reinforced with graphene oxide and bentonite for enhanced adsorption of methylene blue. Carbohydr. Polym..

[44] Liu L., Gao Z.Y., Su X.P., Chen X., Jiang L., Yao J.M. (2015). Adsorption Removal of Dyes from Single and Binary Solutions Using a Cellulose-based Bioadsorbent. ACS Sustain. Chem. Eng..

[45] Seera S.D.K., Kundu D., Gami P., Naik P.K., Banerjee T. (2021). Synthesis and characterization of xylan-gelatin cross-linked reusable hydrogel for the adsorption of methylene blue. Carbohydr. Polym..

[46] Lu B., Lin Q., Yin Z., Lin F., Chen X., Huang B. (2021). Robust and lightweight biofoam based on cellulose nanofibrils for high-efficient methylene blue adsorption. Cellulose.

[47] Zhang X., Lin X., He Y., Luo X. (2019). Phenolic hydroxyl derived copper alginate microspheres as superior adsorbent for effective adsorption of tetracycline. Int. J. Biol. Macromol..

[48] Shenvi S.S., Isloor A.M., Ismail A.F., Shilton S.J., Al Ahmed A. (2015). Humic Acid Based Biopolymeric Membrane for Effective Removal of Methylene Blue and Rhodamine, B. Ind. Eng. Chem. Res..

[49] Li W., Zuo P., Xu D., Xu Y., Wang K., Bai Y., Ma H. (2017). Tunable adsorption properties of bentonite/carboxymethyl cellulose-g-poly(2-(dimethylamino) ethyl methacrylate) composites toward anionic dyes. Chem. Eng. Res. Des..

[50] Zhang P., Ouyang S., Li P., Huang Y., Frost R.L. (2019). Enhanced removal of ionic dyes by hierarchical organic three-dimensional layered double hydroxide prepared via soft-template synthesis with mechanism study. Chem. Eng. J..

[51] Makhado E., Pandey S., Ramontja J. (2018). Microwave assisted synthesis of xanthan gum-cl-poly (acrylic acid) based-reduced graphene oxide hydrogel composite for adsorption of methylene blue and methyl violet from aqueous solution. Int. J. Biol. Macromol..

[52] Boukhalfa N., Boutahala M., Djebri N., Idris A. (2019). Kinetics, thermodynamics, equilibrium isotherms, and reusability studies of cationic dye adsorption by magnetic alginate/oxidized multiwalled carbon nanotubes composites. Int. J. Biol. Macromol..

[53] Wang Y., Pan J., Li Y., Zhang P., Li M., Zheng H., Zhang X., Li H., Du Q. (2020). Methylene blue adsorption by activated carbon, nickel alginate/activated carbon aerogel, and nickel alginate/graphene oxide aerogel: A comparison study. J. Mater. Res. Technol..

[54] Kaur R., Kaur A., Umar A., Anderson W.A., Kansal S.K. (2019). Metal organic framework (MOF) porous octahedral nanocrystals of Cu-BTC: Synthesis, properties and enhanced adsorption properties. Mater. Res. Bull..

[55] Zhang X., Lin X., He Y., Chen Y., Luo X., Shang R. (2019). Study on adsorption of tetracycline by Cu-immobilized alginate adsorbent from water environment. Int. J. Biol. Macromol..

[56] Jeong M., Jang H., Cha H.-J., Park B., Kim J., Yoo J.-K., Park T., Yi J.W., Seong D.G., Oh Y. (2021). The shape tunable gelatin/carbon nanotube wet-gels for complex three-dimensional cellular structures with high elasticity. Carbon.

[57] Yu B., Bai Y., Ming Z., Yang H., Chen L., Hu X., Feng S., Yang S.-T. (2017). Adsorption behaviors of tetracycline on magnetic graphene oxide sponge. Mater. Chem. Phys..

